# Impact of Exercise Modalities on Peripheral and Central Components of Cardiorespiratory Capacity in Heart Transplantation Patients: A Systematic Review and Meta-Analysis

**DOI:** 10.3390/medicina58010032

**Published:** 2021-12-24

**Authors:** Natália Turri-Silva, Francisco Valdez Santos, Wanessa Camilly Caldas Rodrigues, Josuelir Silva Freire, Lawrence C. Cahalin, Kenneth Verboven, João Luiz Quaglioti Durigan, Dominique Hansen, Gerson Cipriano

**Affiliations:** 1 Health and Technologies in Health Sciences Program, University of Brasilia, Brasilia 72220-275, Brazil; natalia.turridasilva@uhasselt.be (N.T.-S.); limasvaldez03@gmail.com (F.V.S.); wanessacamilly@gmail.com (W.C.C.R.); josuelir.sf@gmail.com (J.S.F.); joaodurigan@gmail.com (J.L.Q.D.); ciprianeft@gmail.com (G.C.J.); 2 REVAL/BIOMED—Rehabilitation Research Center, Faculty of Rehabilitation Sciences, Hasselt University, 3590 Diepenbeek, Belgium; kenneth.verboven@uhasselt.be; 3 Heart Centre Hasselt, Jessa Hospital, 3500 Hasselt, Belgium; 4 Department of Education and Training in Oncology, Cancer Institute of São Paulo, São Paulo 03102-002, Brazil; 5 Department of Physical Therapy, University of Miami Miller School of Medicine, Coral Gables, FL 33167-3495, USA; l.cahalin@gmail.com

**Keywords:** exercise, heart transplantation, prognosis, exercise tolerance

## Abstract

*Background and Objectives:* To analyze the effects of aerobic, resistance, and combined training on peripheral and central components related to cardiorespiratory capacity after HTx. *Materials and Methods*: No time restriction was applied for study inclusion. MEDLINE/PubMed; EMBASE, CENTRAL, and PEDro databases were investigated. Studies reporting heart transplanted patients older than 19 years following aerobic, resistance, and combined training according. The outcomes included: V′O_2_ peak, VE/V’CO_2_ slope, heart rate (HR peak), systolic and diastolic blood pressure (SBP and DBP peak), maximum repetition test(1RM), sit-to-stand test, and flow-mediated dilation (FMD). The studies were selected by consensus. Four hundred ninety-two studies initially met the selection criteria. Cochrane handbook was used for abstracting data and assessing data quality and validity. Independent extraction by two observers was applied. *Results*: Isolated aerobic training leads to a greater increase in V′O_2_ peak than combined training compared to the control group (*p* < 0.001, I2 = 0%). However, no significant differences were found in the subgroup comparison (*p* = 0.19, I2 = 42.1%). HR peak increased similarly after aerobic and combined training. High-intensity interval training (HIIT) was better than moderate continuous intensity to increase the V′O_2_ after long term in HTx. Still, there is scarce evidence of HIIT on muscle strength and FMD. No change on VE/V’CO_2_ slope, FMD, and SBP, DBP peak. 1RM and the sit-to-stand test increased after resistance training (*p* < 0.001, I2 = 70%) and CT (*p* < 0.001, I2 = 0%) when compared to control. *Conclusions*: Aerobic and combined training effectively improve VO_2_ peak and muscle strength, respectively. HIIT seems the better choice for cardiorespiratory capacity improvements. More studies are needed to examine the impact of training modalities on VE/V’CO_2_ slope and FMD.

## 1. Introduction

Despite the improvements in pharmaceutical and resynchronization treatments and even considering the advent of the left-ventricular assist device [1], heart transplantation (HTx) remains a notable treatment for advanced heart failure [2,3,4]. HTx gives a new life opportunity for such patients improving peak oxygen uptake (V′O_2_ peak) [5,6,7], a well-recognized prognostic variable assessed via cardiopulmonary exercise test and a gold standard approach to measure sources of exercise limitation in all contest of heart failure. Peak oxygen uptake is still reduced in HTx in comparison to healthy age-matched individuals [8,9]. Nonetheless, to reach a better prognosis after HTx, a V′O_2_ peak increase is wanted [9].

Reduced exercise capacity is associated with cardiac, vascular, and muscular limitations post-HTx [10]. Cardiovascular limitation involves chronotropic incompetence with higher resting heart rate and reduced peak heart rate [10,11]. Peripheral limitations involve vascular endothelial dysfunction by flow-mediated dilation [FMD] reductions [10,12,13,14,15,16,17] and losses in lean mass affecting muscle strength and exercise intolerance post HTx [10]. The immunosuppressive treatment also promotes muscle and V′O_2_ peak reduction [10,18], and peak systolic and diastolic blood pressure increase due to its vasoconstrictor effect [19]. Peak heart rate and systolic blood pressure are also affected by the sympathetic reduction due to the removal of the sinus node [19].

Clinical practice guidelines recommend exercise training for HTx to increase exercise capacity [19]. Regarding exercise training modalities, aerobic training (AT) increases V′O_2_ peak, reduces both systolic and diastolic blood pressure, and perceived exertion in HTx patients [20]. Resistance training (RT) added to aerobic training improves muscle strength and V′O_2_ peak in HTx [21]. Reductions in blood pressure and increments in muscle strength can significantly influence the increase in exercise capacity. VE/V′CO_2_ slope can stratify the survival rate being an important parameter post-HTx.

There is bolding evidence regarding exercise program’s effects post-HTx [9]; however, the pivotal understanding about the preferred exercise prescription for exercise capacity (modality and intensity) remain unexplored. This review evaluates and compares the isolated and combined effect of the aerobic training (AT) and resistance training (RT) on cardiorespiratory components (V′O_2_ peak and VE/V′CO_2_ slope), cardiovascular components (HR peak, SBP peak, and DBP peak), and peripheral components (FMD and muscle strength) post-HTx. We hypothesized that aerobic training with moderate intensity is more favorable post-HTx.

## 2. Materials and Methods

### 2.1. Searches

We followed the recommendations described in the preferred reporting items for systematic reviews and meta-analyses (PRISMA) and Cochrane Handbook [22]. The protocol was registered in the PROSPERO database (https://www.crd.york.ac.uk/prospero/display_record.php?RecordID=59911, accessed on 20 December 2021) under number: CRD42017059911. The systematic review was performed in MEDLINE/PubMed; EMBASE, CENTRAL, and PEDro. The search strategy can be checked on Electronic Supplementary Materials.

### 2.2. Study Inclusion and Exclusion Criteria

Eligibility criteria: (1) Randomized Clinical Trials (RCTs) with or without a cross-over strategy, (2) participants ≥19 years old, who received HTx, (3) studies that described the aerobic training (AT), resistance training (RT), or the combination of both (CT), at any intensity; and (4) studies that compared physical training through exercise with a control group without exercise or comparisons between modalities or training intensities. Language inclusion: English, French, Dutch, and Portuguese. We excluded studies without comparison groups and with aquatic exercise.

### 2.3. Types of Interventions and Outcomes

We considered isolated AT, RT or CT performed at a hospital, outpatient, or home-based setting. We considered exercise interventions post-HTx with the following characteristics: (1) Frequency: at least two days per week; (2) Duration: at least eight weeks and (3) Intensity: at least 50% of maximum heart rate (HRmax) or 50% of V′O_2_ peak for aerobic exercise and 40% of one maximum repetition (1RM) for resistance exercise. The clinical outcomes of the studies should have included at least one of the following measures: peak oxygen uptake (V′O_2_ peak, mL * kg^−1^ * min^−1^), VE/V′CO_2_ slope, peak systolic and diastolic blood pressure (SBP peak and DBP peak, mmHg), peak heart rate (HR peak, bpm), muscle strength (1RM and sitting to stand test) and flow-mediated dilation (FMD, %).

### 2.4. Data Extraction, Synthesis, and Presentation

Type of study, population, interventions (including the type of exercise, intensity, frequency, duration, and modality), comparison and outcomes, risk of bias, and results were extracted. A single researcher performed the data extraction procedure, and a second researcher scrutinized it. All recommendation for a systematic review with metanalysis was followed according to Cochrane Handbook version 6.0 [22]. All analyses were conducted using Review Manager (RevMan) [Computer program]. Version 5.4. The Cochrane Collaboration, 2020. More details about data synthesis description and analysis are on Electronic Supplementary Materials.

Electronic Supplementary Materials also includes Quality of the trials, Risk of bias assessment, and Summary of Findings Table with quality of evidence.

## 3. Results

### 3.1. Selection, Evaluation of Studies, and Quality Assessment

**Figure 1:**
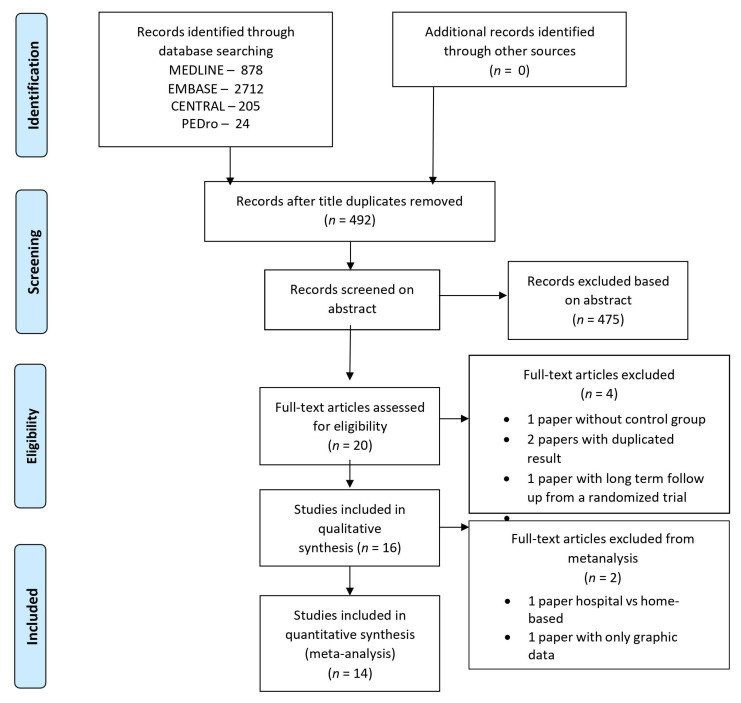
PRISMA Flowchart of the procedures for the selection of articles inserted in the final analysis.

The initial search identified 2712 studies and the present systematic review included 15 studies based on the inclusion criteria, and 13 studies considered for metanalysis. The reasons for exclusion are in Figure 1.

Nine studies involving AT [23,24,25,26,27,28,29,30,31], three studies involving CT [32,33,34], and two involving RT [35,36] were included. Only two studies compared exercise intensities [30,31] and one study compared home-based versus hospital-based intervention [37]. All the other studies included an intervention group compared to a non-exercise control group.

### 3.2. Studies Included in the Systematic Review

The publication period of the included studies ranged from 1998 to 2019, involving a total of 453 patients undergoing HTx with 407 (72%) males, with a mean age of 51 years in the intervention group and 47 years in the control group. Two studies reported only the RT protocol and three studies, included combined training (resistance + aerobic training). Surprisingly, only one of those CT studies reported a detailed prescription of the RT [30]. Also, only two studies presented a comparison between aerobic intensities, high-intensity interval training (HIIT) vs. moderate continuous training (MCT-AE) [31,38], and one study compared hospital-based versus home-based exercise following the same training prescription. Table 1 illustrates the studies summary. PEDRo score is included in Table 1 to provide the quality score from each study individually (widely used in the rehabilitation field).

Regarding the AE, the average frequency was 3 days per week, with a duration of 35 ± 5 min per session and a protocol duration of 18 ± 11 weeks. Among the AE modalities, with MCT-AE, the intensity ranged from 60–80% of V′O_2_ peak [24,28,32], 10% below the anaerobic threshold [23], or Borg RPE between 11–14 [25,29].

One study utilized the higher patient’s tolerance sustained for 30 min [33]. Four studies applied HIIT [26,27,30,31] and one applied HIIT alternating with continuous training [32]. HIIT intensities ranged from 80–90% of the V′O_2_ peak, 80–95% of the HR peak, or 90–100% [32,39] of the baseline peak power output. Interval duration varied from 30 s to 4 min, alternated by low-intensity phases with an intensity ranging from 11 to 13 [27] according to the BORG scale or a recovery rest phase [26,32,39]. Recovery duration varied from 30 s to 3 min, while some studies adopted passive rest recovery.

Heterogeneity was low for V′O_2_ peak, slope VE/V′CO_2_, and sit-to-stand test (I² < 50%). SBP, DBP and HR peak, FMD, and 1RM indicated high heterogeneity (I^2^ > 50%). No study reported adverse effects. The agreement level between the reviewers, by Kappa coefficient, was 0.95 (95% CI: 0.75 to 1.03. 3 Exercise effects on peak oxygen consumption).

**Figure 2:**
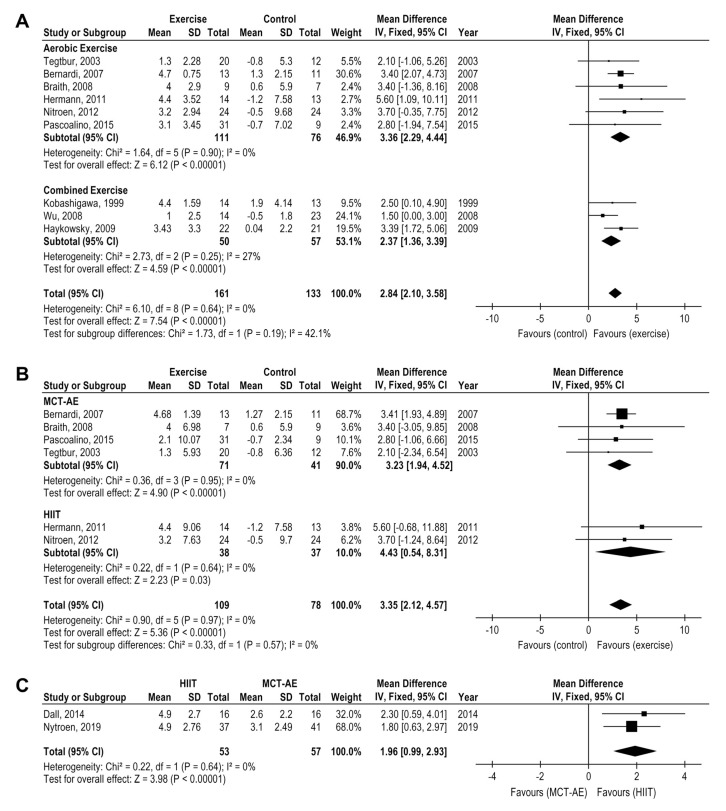
Impact of exercise training on V′O_2_ peak (mL.kg.min) in HTx (**A**) exercise versus the control group (**B**) moderate continuous training and high-intensity training versus the control group (**C**) moderate continuous training versus high-intensity interval training.

Exercise training significantly improve V′O_2_ peak considering all pooled data (9 studies, *n* = 294 patients) with a mean difference [MD] = 2.84, 95% CI: 2.10 to 3.58, mL·kg^−1^·min^−1^, I2 = 0%. However, greater V′O_2_ peak were found for isolated AE(6 studies, *n* = 187 patients) MD = 3.36, 95% CI: 2.29 to 4.44 mL·kg^−1^·min^−1^, I² = 0% than combined intervention (CT) (3 studies, *n* = 107 patients) MD = 2.37, 95% CI: 1.36 to 3.39 mL·kg·min^−1^, I2 = 27% (Figure 2A) when compared to control group.

A greater V′O_2_ peak increase were found for HIIT-AE (2 studies, *n* = 75 patients), MD = 4.43, 95% CI: 0.54 to 8.31 mL·kg·min^−1^, I^2^ = 0%) than MCT-AE (4 studies, *n* = 112 patients), MD = 3.23, 95% CI: 1.94 to 4.52 mL·kg·min^−1^, I^2^ = 0%, (Figure 2B) when both were compared to a control group. The HIIT superiority over MCT-AE was directly demonstrated when compared one versus another (2 studies, *n* = 110 patients) with a mean difference MD = 1.96, 95% CI: 0.99 to 2.93 mL kg^−1^ min^−1^, I² = 0% (Figure 2C).

### 3.3. Exercise Effects on Peak Heart Rate

**Figure 3:**
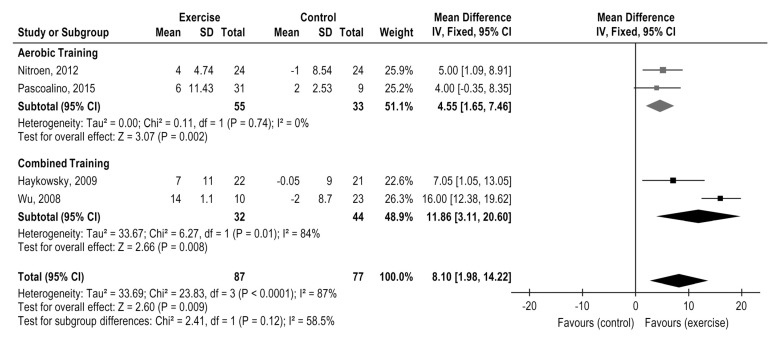
Impact of exercise training on Heart Rate peak (bpm) in HTx: exercise versus the control group.

The analysis of peak heart rate was separated according to time post-HTx: de novo and long therm. Comparing the pooled effect analysis of aerobic and combined training versus control group after long term post HTx (≥1 year), jointly both training induced a slightly favorable effect in HR peak (4 studies, *n* = 164 patients), MD = 8.10, 95% CI: 1.98 to 14.22 bpm, I^2^ = 87%, *p* = 0.009, with no subgroup differences (*p* = 0.12) (Figure 3.)

Two studies have explored exercise versus control in de novo HTx (˂1 year) and both aerobic [29] and combined training [33] did not indicate improvement on HR peak. Two studies compared HIIT versus moderate continuous training in de novo [30] and long term [31] post-HTx and the delta comparison indicated better results for HIIT only in long term post-HTx (*p* = 0.027) [31].

### 3.4. Exercise Effects on Peak Systolic and Diastolic Blood Pressure

**Figure 4:**
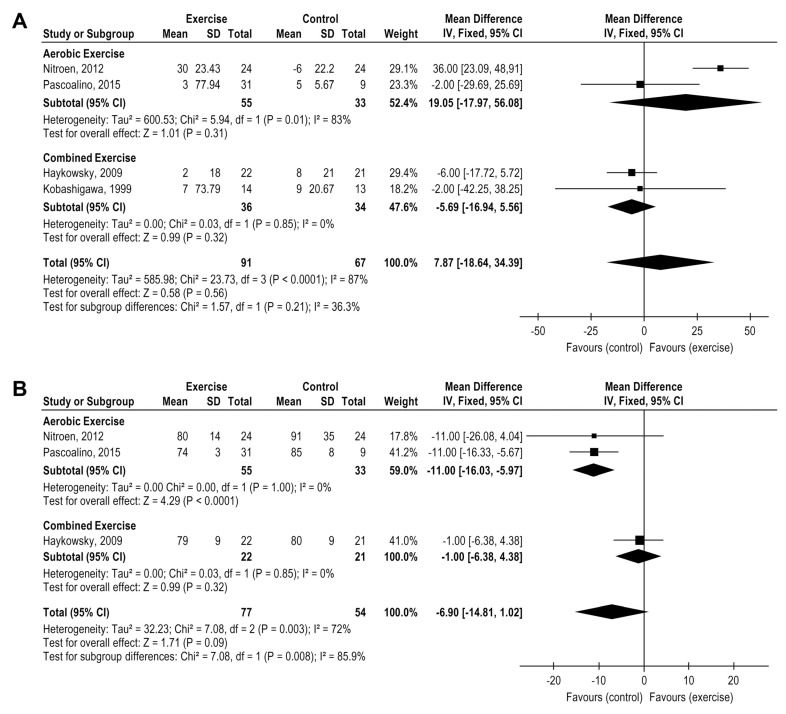
Impact of exercise training on blood pressure (mmHg) in HTx (**A**) exercise versus control group for systolic blood pressure (**B**) exercise versus control group for diastolic blood pressure.

Aerobic and combined exercise modalities comparison did not demonstrate changes in SBP peak post-HTx (4 studies, *n* = 158 patients), MD = 7.87, 95% CI: −18.64 to 34.39, mmHg, I^2^ = 87% (Figure 4A) and DBP peak (3 studies, *n* = 131), MD = −6.90, 95% CI: −14.81 to 1.02, mmHg, I^2^ = 72% (Figure 4B) although the separated analysis demonstrated a superior effect on DBP peak reduction from the aerobic exercise compared with a control (2 studies, *n* = 135), MD = −11.0, 95% CI: −16.03 to −5.97 mmHg, I^2^ = 0% (Figure 4B).

### 3.5. Exercise Effects on VE/V′CO_2_ Slope

**Figure 5:**
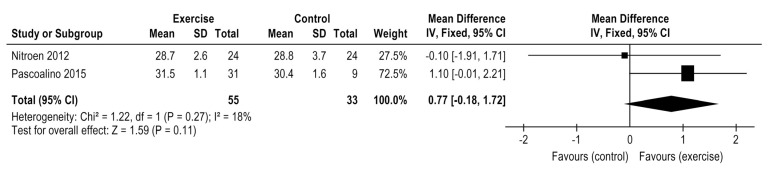
Impact of exercise training on VE/V′CO_2_ slope in HTx.

**Figure 6:**
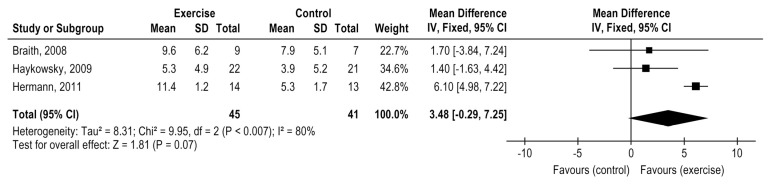
Impact of exercise training on flow-mediated dilation in HTx.

Only two studies had reported this outcome comparing exercise versus control group in HTx patients, both involving aerobic training [27,28]. The exercise treatment did not demonstrate any difference on VE/V′CO_2_ slope (2 studies, *n* = 88 patients), MD = 0.77, 95% CI: −0.18 to 1.72, I^2^ = 18%, *p* = 0.11 (Figure 5 and Figure 6). One study explored the comparison between HIIT vs moderate continuous training, not indicating any differences between them (*n* = 78), MD = −1.6 (−5.2 to 2.0), *p* = 0.375 [30].

### 3.6. Exercise Effects on Flow-Mediated Dilation

Three studies compared the exercise treatment with control in HTx patients. Exercise training did not demonstrate positive effect on FMD (3 studies, *n* = 86 patients), MD = 3.48%, 95% CI: −0.29 to 7.25%, *p* = 0.07). However, the studies presented a high heterogeneity (I² = 80%)-Figure 6. From those included studies, one study applied MCT-AE [25], one study applied CT [32], and one HIIT [26]. Only HIIT [28] presented an expressive improvement in FMD. However, a subgroup analysis was not possible due to the small number of studies in each exercise modality. Nytroen 2019 compared HIIT vs moderate continuous training and did not indicated differences for this parameter between modalities (*n* = 78), MD = −1.5 (−4 to 0.9), *p* = 0.208 [30].

### 3.7. Exercise Effects on Muscle Strength

**Figure 7:**
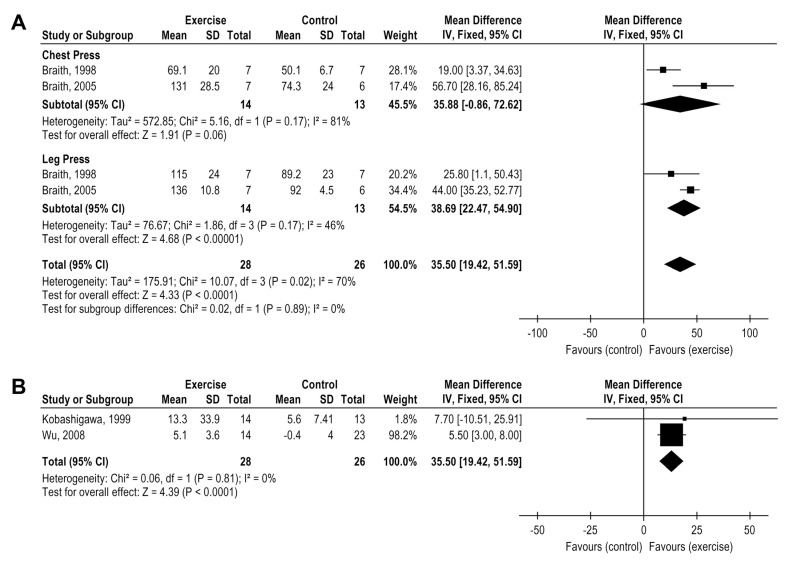
Impact of resistance training on muscle strength in HTx (**A**) 1 maximum repetition test (**B**) sit-to-stand test.

Six studies analyzed the exercise impact on muscle strength, but two were not included in the metanalysis forest plots due to different muscle strength assessments (Isokinetic and isotonic evaluations). Two studies evaluated the maximum repetition test (1RM) and two evaluated the sit-to-stand test. Isolated resistance training (RT) was associated with a significant improvement in the 1RM for both chest press and leg extension movements, MD = 35.50 Kg, 95% CI: 19.42 to 51.59, I² = 70, *p* < 0.0001) (Figure 7A) while the other two studies involving combined training showed increases on the sit-to-stand test, MD: 5.54, 95% CI 3.07 to 8.01; I² = 0% (Figure 7B). Isokinetic and isotonic evaluations not included in the metanalysis forest plots, also suggested an increase in muscle strength after CT and AT, respectively [27,34].

## 4. Discussion

The novelty of this meta-analysis is the analysis of different exercise modalities and intensities in clinical outcomes related to cardiorespiratory capacity after HTx, strengthening information about training prescription for clinical practice. A superior effect of aerobic training to improve V′O_2_ peak in HTx patients was demonstrated with a moderate level of certainty of evidence. Interesting, high-intensity interval training (HIIT) demonstrated a higher effect on the V′O_2_ peak than moderate continuous training (MCT-AE) with no adverse effect [30,31].

Considering the low level of certainty of the evidence, resistance exercise training (RT) led to improvements in muscle strength. The skeletal muscle weakness, vasodilatory capacity impairment, and muscle capillary density reduction are the main peripheral factors related to exercise capacity reductions after HTx and partially explain the V′O_2_ peak impairment [40]. Nytrøen et al. pointed to the association between muscular deconditioning and V′O_2_ peak reduction [27], recognizing the peripheral limitations in HTx patients. Peripheral adaptation such as mitochondrial volume density, oxidative enzyme capacity, and the percentage of type 1 muscle fibers distribution increase, are associated with the cardiorespiratory capacity increase [10,36,41]. These results indicate RT, isolated, or in combination with AT, increases muscle strength and attenuates V′O_2_ peak impairment post-HTx.

There was no evidence that exercise affect DBP and SBP peak in HTx [27,28,33] and like HR peak, all indicated a very low level of certainty of evidence. HR peak increased after all training modalities, especially after AE. Compared to MCT-AE, a higher increase in HR peak occurred after HIIT with a moderate level of certainty of evidence. However, the magnitude seems not equivalent to the exercise capacity improvement [6,42,43], possibly due to the chronotropic incompetence [24,44]. Autonomic nervous system improvement may explain it [24,43,44]. The average increase of SBP peak should be 50% of the resting value and an insufficient increase has been associated with left ventricular systolic dysfunction [45]. Nevertheless, the relationship between SBP response and V′O_2_ peak is unclear.

The absence of improvements after CT [33], HIIT [27,46], or MCT-AE [47] on LV end-diastolic or end-systolic volume, stroke volume, or ejection fraction after HTx [10] contributes to the rationale that the improvements in peak V′O_2_ seem not only related to central (cardiac) adaptations [10].

A healthy endothelium function positively impacts exercise-induced vasodilation capacity, an essential part of the maintenance of adequate V′O_2_ during exercise. FMD increase positively influences V′O2 peak in healthy individuals [48,49,50], coronary heart disease, hypertension, and heart failure [51,52,53,54]. Inversely, endothelial dysfunction is associated with plaque progression and a lower peak V′O2 post-HTx [55,56].

Exercise training was not associated with FMD benefits [25,26,32] (very low level of certainty of the evidence), but an expressive improvement was when HIIT was compared to a control group [26]. More studies are needed, but recognizing that endothelial dysfunction predicts cardiac allograft vasculopathy [14], HIIT seems a promising approach post-HTx. The unique study that compared HIIT vs. moderate continuous training did not indicate differences between those modalities, although the statistical difference was seen only within the HIIT group. Although an endothelial function recovery occurs post-HTx [57], peripheral endothelial dysfunction remains after 1 to 13 years [58]. The primary mechanism of the endothelial dysfunction post-HTx relates to cyclosporin therapy [10,57]. Exercise training can counteract it by enhancing nitric oxide (NO) production [48,49].

According to the two included studies and with a very low level of evidence, there is no effect on VE/V′CO_2_ slope after exercise training post-HTx when compared to a control group. Nytroen, 2019 when comparing HIIT vs. moderate continuous training also did not reveal any difference between modalities [30]. VE/V′CO_2_ slope is a strong independent predictor of mortality in HF patients [59] as accurate as V′O_2_ peak for HTx. Although the VO_2_ peak is related to mortality like VE/VCO_2_ slope, none of the included studies addressed morbidity or mortality rates after their intervention or control period. The positive association between VE/V′CO_2_ slope reduction and functional capacity improvement was identified in 40% of the patients post-HTx, even five years later [60]. VE/V′CO_2_ slope increase has been associated with peripheral factors, such as muscle deconditioning, peripheral oxygen transport problems, and type IIb-muscle-fiber increased, leading to a primary lactic acidosis during exercise demanding high ventilatory response [60]. In our meta-analysis, only aerobic training (27) (28) explored VE/V′CO_2_ slope post-HTx.

The small number of studies available and the low level of certainty of evidence from many outcomes is the major limitation of this systematic review with meta-analysis. Additionally, the lack of studies reporting comparison between modalities has limited the results of exercise training post-HTx. More research is required, mainly for the outcomes VE/V′CO_2_ slope and FMD. In addition, the absence of an increase in heart rate peak after exercise training may have been influenced by the poor autonomic response due to cardiac denervation. Another limitation is the absence of information regarding the age mismatch between donors and recipients of the included studies. Finally, it is also a problem that patients with left ventricular assistant systems before transplantation often present good exercise tolerance and are not scrutinized from those without left ventricular assistant systems [61] which could influence the final meta-analysis results. However, even considering the limitations, this review is important to demonstrate the state of the art on training prescription in HTx, revealing the need for new clinical trials with higher quality.

## 5. Conclusions

Cardiac rehabilitation is essential after HTx to improve the training performance of the patients. From this review, aerobic training seems the best training modality after HTx, mainly high-intensity interval training with the biggest effect on peak oxygen consumption 7265677. Isolated resistance training or combined training improve muscle strength. More studies are needed.

## Figures and Tables

**Table 1:** Description of studies included in the systematic review.

Study^ref^, Year, Type		Time after HTx and Local	Sample Size (*n*)		Age (yrs) (mean ± SD)	Outcomes	Intervention Description	Frequency (d/wk)	Session Duration (min)	Program Duration (wk)	PEDro Score
AEROBIC TRAINING VS. CONTROL											
Tegtbur [23] RCT	2003	5 years	AT	20	55.0 ± 7.0	VO2_peak_	Outpatient–home-based-controlled remotely AT: bicycle ergometer (10% below the Anaerobic threshold); CG: usual medical care	3	28	48	4
			CG	12	54.0 ± 8.0						
Bernardi et al. [24] RCT	2007	6 months	AT	13	34.9 ± 4.0	VO2_peak_	Outpatient–home-based–non-supervised AT: bicycle ergometer (50 rpm for 30 min at 60–70% of VO2_peak._ New training load calculated after 3 months by a new exercise test to exhaustion); CG: avoid exercise above their regular pre-study routine and specifically to avoid exercise that would lead to feelings of dyspnoea or exhaustion.	5	30	24	4
			CG	11	33.9 ± 4.3						
		
Pierce et al. [29] RCT	2008	8 weeks	AT	08	53.6 ± 13.6	VO2_peak_, HR_peak_	Outpatient–clinic-supervised. AT: Training protocol started with 30 min of continuous exercise and progressed to 34 to 40 min as tolerated after the initial 4 weeks. Continuous treadmill walking (Borg RPE between 11–13, or ‘moderate’ to ‘somewhat hard’ range, following ACSM guidelines. Progression to an RPE in the 12–14 Borg scale range ‘as tolerated’ by each participant) CG: standard medical care and encouragement to engage in regular walking, but did not participate in a supervised exercise.	-	Initial: 30 After 4 wk: 40	12	4
			CG	06	54.2 ± 6.4						
Braith et al. [25] RCT	2008	8 weeks	AT	09	54.3 ± 9.5	VO2_peak,_ FMD	Hospital-supervised AT: initial 4 weeks: 5 min warm-up + 30 min continuous treadmill walking + 5 min cool-down. Exercise progressed to 35 to 40 min thereafter. The intensity in a range from Borg RPE between 11–13, or ‘moderate’ to ‘somewhat hard’ range and progressing to RPE in the 12–14 Borg scale range ‘as tolerated’ following ACSM guidelines. CG: standard medical care and encouragement to engage in regular walking but did not participate in a supervised exercise.	3	Initial: 30 After: 35 to 40 as tolerated	12	5
			CG	07	54.4 ± 13.1						
Hermann et al. [26] RCT	2011	1 year	AT	14	53.0 ± 11.0	VO2_peak_, FMD	Outpatient–clinic-supervised. AT: warm-up (above 50% VO_2_peak) + HIIT on bicycle (interval blocks of 4 min/2 min/30 s according to 80%, 85%, and 90% of VO_2 peak_ and 30 s recovery periods) + staircase running (80% of peak VO_2 peak_); CG: Patient education (4 h of teaching to the patients about the benefits of exercise training together with information on nutrition)	3	42	8	7
			CG	13	47.0 ± 18.0						
Nytrøen et al. [27] RCT	2012	1–8 years	AT	24	48.0 ± 17.0	VO2_peak,_ HR_peak,_ SBP_peak_ DBP_peak_, VE/VCO_2_ slope, Muscle strength	Outpatient–clinic-supervised. AT: HIIT on a treadmill (10 min warm-up + 4 min exercise bouts at 85–95% of HR_peak_, separated by 3 min active pauses at Borg scale 11–13, 6–20 RPE). Additionally, the patients were encouraged to continue any physical activity on their own. CG: No intervention was given to the control group other than basic.	3	35	24	5
			CG	24	53.0 ± 14.0						
Pascoalino et al. [28] RCT	2015	≥1 year	AT	31	45.0 ± 3.0	VO2_peak,_ VE/VCO_2_ slope, HR_peak,_ SBP_peak,_ DBP_peak_	Outpatient–clinic-supervised. AT: Supervised: 5 min warm-up + 40 min walking/jogging on a treadmill (80% HR of the RCP - 69.0% ± 1.9 % of VO_2 max_. Endurance Exercise Intensity was continually adjusted) + 5 min cool down. Non-supervised: Same exercise protocol following exercise intensity of 11–13 on the rate of Borg scale (range: 16–20); CG: maintain their daily activities without AE during the 12-week period.	3	40	12	6
			CG	09	45.0 ± 6.0						
COMBINED TRAINING VS. CONTROL GROUP											
Kobachigawa et al. [33] RCT	1999	2 weeks	CT	14	55.0 ± 8.0	VO2_peak_, HR_peak,_ SBP_peak,_ Muscle strength	Outpatient–clinic-supervised. RT: closed-chain resistive activities + abdominal exercises; AT: Treadmill or bicycle ergometer (a goal of at least 30 min of continuous exercise at a moderate intensity according to patient’s tolerance) CG: Written guidelines (exercises at home)	1–3	AT: ≥30	24	5
			CG	13	50.0 ± 12.0						
		
Wu et al. [34] RCT	2008	1 year	CT	12	60.6 ± 6.2	VO2_peak_, HR_peak,_ Muscle strength	Outpatient–home-based–supervised every 1–2 weeks. RT: 5 min warm-up + upper and lower extremity light-weight; AT: 15–20 min walking at a prescribed intensity with 60–70% VO_2_ peak + stepping exercise with a stool + 5 min cool down. CG: control group was asked to keep their usual activity lifestyle during the study period.	3	40	8	5
			CG	19	51.6 ± 12.8						
		
Haykowsky et al. [32] RCT	2009	≥0.5 year	CT	22	57.0 ± 11.0	VO2_peak_, HR_peak,_ SBP_peak,_ DBP_peak_, FMD	Outpatient–hospital-supervised. AT: treadmill and bicycle (HR: 60–80% VO_2 peak_) for 30–45 min. After 4 weeks, continuous aerobic training 3 days/week (HR = 80% VO_2 peak_) + Interval training 2 days/week (10 to 25 rep–gradually increase-of 30 s exercise at 90–100% baseline peak power output followed by 60 s rest); RT: upper (chest press, latissimus dorsi pulldown, arms curls) and lower extremity (leg press) strength training 10 rep, gradually increased until 25 rep at 50% of maximal strength (1 rep = 30 s exercise and 60 s rest); CG: continued with their usual activities of daily living.	AT: 5 RT: 2	30–45 45	12	4
			CG	21	57.0 ± 11.0						
		
RESISTANCE TRAINING VS. CONTROL GROUP											
Braith et al. [35] RCT	1998	2 months	RT	7	54 ± 3	Muscle strength	Outpatient–clinic–supervised. RT: 5 min of warm-up walking on a treadmill + lumbar extensor training 1 day/week and upper and lower body resistance training 2 days/week. A single set of 10–15 repetitions was completed for each exercise: lumbar extension, duo-decline chest press, knee extension, pullover, knee flexion, triceps extension, biceps flexion, shoulder press, and the abdominal machine. The initial training weight represented 50% of the one-repetition maximum (1-RM) test. The transplant recipients were not permitted to exceed 15 repetitions. Rather, when 15 repetitions were successfully achieved, the weight was increased by 5–10% at the next training session. CG: No resistance training intervention	3	Not described	12 24	4
			CG	7	51 ± 8						
Braith et al. [36] RCT	2005	2 months	RT	8	52 ± 2	Muscle strength	Outpatient–home-based-supervised RT: standard care home-based walking program (not supervised) associated with resistance training. 5 min warming-up + a single set from 10 to 15 repetitions were completed for each exercise: chest press, knee extension, pulldown, seated leg curl, shoulder press, seated triceps dip, biceps curl, and lumbar extension at 50% of 1 RM. The resistance was increased by 5% to 10% at the next training session when 15 repetitions were successfully achieved. Upper body exercises were alternated with lower body exercises. CG: standard care home-based walking program (not supervised)	2	Not described	24	6
			CG	7	53 ± 2						
HIIT VS. MCT											
Dall [31] RCT	2014	≥1 year	HIIT MCT	16	51.9 (33–70) Cross-over	VO2_peak_, HR_peak_	Outpatient–clinic-supervised. AT–HIIT: Each HIIT session consisted of 16 min interval training with intervals of 4-, 2-and1-min duration at >80% of VO2peak, separated by a 2-min active rest period (approx. 60% of VO2peak)	3	16 45	12	7
							AT–MCT-AE: The CON sessions consisted of biking for 45 min with an intensity corresponding to 60–70% of VO2peak. All sessions began with a 10 min warm-up and ended with a 10-min cooldown.				
Nytrøen [30] RCT	2019	11 weeks	HIIT	37	50 ± 12	VO2_peak,_ HR_peak,_ VE/VCO_2_ slope, Muscle strength, FMD	Outpatient–clinic-supervised. AT; HIIT: 2- to 4-min intervals at 85% to 95% of peak effort (85%–95% of peak HR or ≈81–93% of Vo2peak-16 to 18 on Borg scale). 3–6 months after HTx, training consisted of 1 HIT session, 1 resistance training session (core musculature and large muscle groups), and 1 combined session per week. From 6–9 months after HTx, 2 HIT sessions and 1 resistance training session per week. The last 2 to 3 months of the intervention consisted of 3 HIT sessions per week	2–3	40	48	7
			MCT	41	48 ± 14		AT; MCT-AE: 60% to 80% of peak effort, regular core strengthening exercises, and exercises for large muscle groups				
HOSPITAL VS. HOME-BASED											
					45.27 ± 13.10 35.61 ± 12.91		CT–Hospital-supervised: Exercise sessions included flexibility exercises, aerobic exercises, strengthening exercises, breathing exercises, and relaxation exercises. 30 min of aerobic exercises on either a treadmill or a stationary bicycle at 60% to 70% of the maximal VO_2_ and a level of 13 to 15 on the Borg scale. After 2 weeks, strengthening exercises were added: abdominal, upper limb, and lower limb muscle groups, using progressively heavier "light-weights", ranging from 250 to 500 g. In the end, all patients performed relaxation exercises according to the Jacobson technique of progressive muscle relaxation				
Karapolat [38] RCT	2007	15 months	CT Hosp CT Home	15 13		VO2_peak_	CT–Home-based–non-supervised: All exercises taught to group CT-Hosp patients were the same ones as those performed by the patients in CT–Home-based group. In addition, a walking program was performed.	3	90	8	3

Legends: HTx: Heart Transplantation; AT: Aerobic Training; RT: Resistance Training; CT: Combined Training; CG: Control Group; VO_2 peak_: peak oxygen uptake; HR_peak__:_: peak heart rate; SBP_peak_: peak systolic blood pressure; VE/VCO_2_ slope: exercise ventilatory efficiency slope; FMD: Flow-mediated dilation; HIIT: High-intensity interval training; MCT-AE: moderate continuous training HRmax: Maximum Heart Rate; RMT: Respiratory Muscle Training; RPC: Respiratory Point Compensation; RPE: Rating of perceived exertion scale; ACSM: American college of sports medicine.

## Data Availability

The data presented in this study are available on request from the corresponding author.

## Supplementary Materials

The following are available online at https://www.mdpi.com/article/10.3390/medicina58010032/s1, Figure S1: Risk of bias assessed via Rob 2 tool, Figure S2: Summary of Findings Tables with quality of evidence for exercise training compared to control, Figure S3: Summary of Findings Tables with quality of evidence for HIIT compared to moderate continous training, Table S1: Physiotherapy Evidence Database (PEDro) scores for each of the 12 included studies in the metanalysis.
