# Supplementary material for: Impact of Exercise Modalities on Peripheral and Central Components of Cardiorespiratory Capacity in Heart Transplantation Patients: A Systematic Review and Meta-Analysis
Source: Medicina (Kaunas). 2021 Dec 24;58(1):32. doi: 10.3390/medicina58010032 (PMC8779927; doi:10.3390/medicina58010032)
Supplement: Supplementary file 1 [file medicina-58-00032-s001.zip › medicina-1461636-supplementary.pdf]

Supplementary file

Search strategy

The search strategy included the cross-checking of the keywords, according to the Medical Subjects Headings (Mesh), from the United States National Library of Medicine. We chose the keywords following the intervention and population criteria and included filters to limit the search only to randomized clinical trials. There was no language restriction. The full electronic search strategy followed the keywords: (i) population: "Heart-Lung Transplantation"[Mesh] OR "Heart Transplantation"[Mesh] (ii) intervention: "Warm-Up Exercise"[Mesh] OR "Cool-Down Exercise"[Mesh] OR "Plyometric Exercise"[Mesh] OR "Exercise Muscle Stretching Exercises"[Mesh] OR "Exercise"[Mesh] OR "Physical Therapy"[Mesh] OR "Exercise Tolerance"[Mesh] OR "Exercise Therapy"[Mesh] OR "Resistance Training"[Mesh] OR "Physical Therapy Specialty "[Mesh] OR "Circuit-Resistance Training"[Mesh] OR "High-Intensity Interval Training"[Mesh]).

The research was limited to Randomized Controlled Trials (RCTs) in humans above 19 years old in the following languages: Dutch, English. French and Portuguese.

The studies were selected according to the Cochrane handbook (25). The authors initially assessed the title and abstract for screening, followed eligibility criteria, including the type of study design, description of the population, and information on interventions.

Quality of the trials

Quality assessment was performed by two independent reviewers (WCCR and JSF) (and a third reviewer (GCJ) in case of disparities). PEDro scale was used (Table and detailed Pedro score on Electronic Supplemental Material Table S1). On this scale, 11 questions were answered as 'yes' (score 1) or 'no' (score 0) to obtain more information about the internal and external validity of the study and interpretability of the statistical results (Pedro). According to Pedro guidelines, item 1 from the Pedro scale was not used to calculate the score, which resulted in a total score maximum of 10.

According to the Pedro scale, specific quality of the trials information per domain is below in Supplementary table 1.

Table S1 Physiotherapy Evidence Database (PEDro) scores for each of the 12 included studies in the metanalysis.

	Study quality criteria									
Study	2	3	4	5	6	7	8	9	10	11	Total
Braith et al. (2008)	1	0	1	0	0	1	0	0	1	1	5
Bernardi et al. (2007)	1	0	1	0	0	0	0	0	1	1	4
Haykowsky et al. (2009)	1	0	0	0	0	0	1	0	1	1	4
Hermann et al. (2011)	1	1	1	0	0	1	1	0	1	1	7
Kobachigawa et al. (1999)	1	1	1	0	0	0	1	0	1	0	5
Nytrøen et al. (2012)	1	0	1	0	0	0	1	0	1	1	5
Pascoalino et al. (2014)	1	0	1	0	0	1	1	0	1	1	6
Pierce et al. (2008)	1	0	1	0	0	0	0	0	1	1	4
Tegtbur et al. (2003)	1	0	1	0	0	0	0	0	1	1	4
Wu et al. (2008)	1	0	1	0	0	0	0	1	1	1	5
Braith et al. (1998)	0	0	1	0	0	1	0	0	1	1	4
Braith et al. (2005)	1	0	1	0	0	1	1	0	1	1	6
Dall et al. (2014)	1	1	1	0	0	1	1	0	1	1	7
Karapolat et al. (2007)	1	0	1	0	0	0	0	0	1	0	3

1 = criterion is satisfied; 0 = criterion not satisfied

Supplementary Figure S1: Risk of bias assessed via Rob 2 tool

THE PER-PROTOCOL EFFECT									
**Study**	**Experimental**	**Comparator**	**Outcome**	**D1**	**D2**	**D3**	**D4**	**D5**	**Overall**
Bernardi 2007	MCT-AE	Control Group	VO2 peak	+	?	+	+	?	!
Rustad 2014	HIIT	Control Group	VO2 peak	+	+	+	?	+	−
Rustad 2014	HIIT	Control Group	HR peak	+	?	+	?	+	!
Nitroen 2012	HIIT	Control Group	VO2 peak	+	+	+	?	+	!
Nitroen 2012	HIIT	Control Group	HR peak	+	+	+	?	+	!
Nitroen 2012	HIIT	Control Group	Peak SBP	?	+	+	−	+	−
Nitroen 2012	HIIT	Control Group	Peak DBP	?	+	+	−	?	!
Nitroen 2012	HIIT	Control Group	VE/VCO2 slope	?	+	+	+	+	!
Monk-Hansen 2015	HIIT	Control Group	VO2 peak	+	−	+	?	+	−
Monk-Hansen 2015	HIIT	Control Group	HR peak	+	−	+	?	+	−
Pascoalino 2015	MCT-AE	Control Group	VO2 peak	?	?	+	?	+	!
Pascoalino 2015	MCT-AE	Control Group	VE/VCO2 slope	?	?	+	?	+	!
Pascoalino 2015	MCT-AE	Control Group	SBP max	?	?	+	?	+	!
Pascoalino 2015	MCT-AE	Control Group	DBP max	?	?	+	?	+	!
Pascoalino 2015	MCT-AE	Control Group	HR peak	?	?	+	+	+	!
Hermann 2011	HIIT	Control Group	VCO2 peak	+	?	+	?	+	!
Hermann 2011	HIIT	Control Group	FMD	?	+	+	+	+	!
Haykowsky 2009	Combined Training	Control Group	VCO2 peak	?	+	+	?	+	!
Haykowsky 2009	Combined Training	Control Group	HR peak	+	+	+	?	+	!
Haykowsky 2009	Combined Training	Control Group	Peak SBP	?	+	+	+	+	!
Haykowsky 2009	Combined Training	Control Group	Peak DBP	?	+	+	+	+	!
Haykowsky 2009	Combined Training	Control Group	FMD	?	?	+	+	+	!
Braith 2008	MCT-AE	Control Group	Peak VO2	?	+	+	?	+	!
Braith 2008	MCT-AE	Control Group	FMD	?	+	+	+	+	!
Kobashigawa 1999	Combined Training	Control Group	VCO2 peak	−	+	+	?	+	−
Kobashigawa 1999	Combined Training	Control Group	VE/VCO2 slope	−	+	+	?	+	−
Kobashigawa 1999	Combined Training	Control Group	HR peak	−	+	+	?	+	−
Kobashigawa 1999	Combined Training	Control Group	sit-to-stand	−	+	+	?	+	−
Kobashigawa 1999	Exercise Training	Control Group	Peak SBP	−	+	+	?	+	−
Braith 1998	Resistance Training	Control	1RM	+	+	+	+	+	!
Braith 2005	Resistance Training	Control	1RM	?	+	+	+	?	!
Dall 2014	HIIT	MCT-AE	VCO2 peak	+	+	+	+	?	!
Dall 2014	HIIT	MCT-AE	HR peak	+	+	+	+	?	!
Nytroen 2019	HIIT	MCT-AE	VCO2 peak	?	+	+	?	?	!
Nytroen 2019	HIIT	MCT-AE	HR peak	?	+	+	?	?	!
THE INTENTION-TO-TREAT EFFECT									
**Study**	**Experimental**	**Comparator**	**Outcome**	**D1**	**D2**	**D3**	**D4**	**D5**	**Overall**
Wu 2008	Combined Training	Control Group	VO2 peak	?	+	+	+	?	!
Wu 2008	Combined Training	Control Group	HR peak	?	+	+	+	?	!
Wu 2008	Combined Training	Control Group	sit-to-stand	?	+	+	+	?	!

+ Low Risk

? Some concerns

− High risk

D1 Randomisation process
D2 Deviations from the intended intervention
D3 Missing outcome data
D4 Measurement of the outcome
D5 Selection of the reported result

Supplementary Figure S2: Summary of Findings Tables with quality of evidence for exercise training compared to control.

Author(s):
Question:
Setting: Exercise training compared to Control after Heart Transplantation
Bibliography: Turri-Silva et al, 2020

Certainty assessment							№ of patients		Effect		Certainty	Importance
№ of studies	Study design	Risk of bias	Inconsistency	Indirectness	Imprecision	Other considerations	Exercise	Control	Relative (95% CI)	Absolute (95% CI)		
**FMD (%)**												
3	randomised trials	not serious	very serious [a]	serious [b]	serious [c]	none	38	36	-	MD **3.48 higher** (0.29 lower to 7.25 higher)	⊕◯◯◯ VERY LOW	
**Ventilatory Equivalent slope (VE/VCO2)**												
3	randomised trials	serious [d]	serious [e]	serious [f]	very serious [g]	none	55	33	-	MD **0.77 higher** (0.18 lower to 1.72 higher)	⊕◯◯◯ VERY LOW	
**Oxygen Uptake (VO2 peak)**												
11	randomised trials	serious [h]	not serious	not serious [i]	not serious [j]	none	204	161	-	MD **2.88 higher** (2.15 higher to 3.61 higher)	⊕⊕⊕◯ MODERATE	
**Oxygen Uptake (VO2 peak) - Aerobic Exercise**												
8	randomised trials	serious [h]	not serious	not serious	not serious [j]	none	146	112	-	MD **3.44 higher** (2.39 higher to 4.49 higher)	⊕⊕⊕◯ MODERATE	
**Oxygen Uptake (VO2 peak) - Combined exercise**												
3	randomised trials	not serious	not serious	not serious	not serious [j]	none	58	49	-	MD **2.36 higher** (1.35 higher to 3.37 higher)	⊕⊕⊕⊕ HIGH	
**Peak Systolic Blood Pressure (mmHg)**												
4	randomised trials	not serious	very serious [a]	serious [k]	very serious [l]	none	91	67	-	MD **7.87 higher** (18.64 lower to 34.39 higher)	⊕◯◯◯ VERY LOW	
**Peak Heart Rate**												
8	randomised trials	serious [m]	very serious [a]	not serious	serious [n]	none	141	120	-	MD **5.41 higher** (0.19 higher to 10.63 higher)	⊕◯◯◯ VERY LOW	
**Maximum Repetition (1 RM)**												
2	randomised trials	not serious	serious [o]	not serious	serious [p]	none	28	26	-	MD **35.5 higher** (19.42 higher to 51.59 higher)	⊕⊕◯◯ LOW	
**Sit-to-stand Test**												
2	randomised trials	not serious	serious [q]	not serious	serious [p]	none	28	36	-	MD **5.54 higher** (3.07 higher to 8.01 higher)	⊕⊕◯◯ LOW	
**Peak Diastolic Blood Pressure (mmHg)**												
3	randomised trials	not serious	very serious [a]	not serious	serious [r]	none	79	56	-	MD **6.9 lower** (14.81 lower to 1.02 higher)	⊕◯◯◯ VERY LOW	

CI: Confidence interval; **MD:** Mean difference

Explanations

a. All criteria for inconsistency were violated. The authors believe it can be influenced by different exercise prescriptions (modalities and training intensities).
b. Indirectness can be related to differences in intervention, population or outcomes measurements. Along those 3 included stuies, Braith 2007 applied aerobic training with moderate intensity, Haykowsky 2009 applied combined training (AER+RES) and Hermann 2011 appliedd HIIT modality. However, there were no relevant variations regarding the assessments and matched population that could have influenced the external validity.
c. Considering not dicotomic outcomes, all data from imprecision were based on confidence interval and sample size. Braith, 2008 and Haykowsky, 2009 presented both positive and negative results (crossed neutral line). Furthermore, considering the small general sample size the authors rate down for imprecision.
d. Unclear randomization and allocation concealed procedure for Bernardi, 2007. Also, although a detailed description of randomization procedure in Pascoalino, 2014, there is a big difference in relation to sample size in between intervention group and control group. Considering these limitations the authors believe there is a serious limitation for VE/VCO2 evidence.
e. Although low heterogeneity, points estimates varied widely across the 2 included studies and showed minimal overlap.
f. Indirectness can be related to differences in intervention. Just 2 studies were included for this outcome and they varied regarding the intervention duration, Nitroen 2012 did the double time of intervention than Pascoalino 2014 (24 vs 12 weeks, respectively). Also, Nitroen 2012 applied HIIT intervention, while Pascoalino 2014 used aerobic moderate intensity.
g. Considering not dicotomic outcomes, all data from imprecision were based on confidence interval and sample size. Considering just 2 studies included the outcome VE/VCO2 slope presenting wide variation on their confidence interval with positive and negative results, the authors rate down for imprecision
h. There are 5 studies considering with serious risk of bias and 1 study with very serious risk of bias. All them without a clear randomization procedure and allocation concealed (Pierce, 2007; Pascoalino, 2014; Braith, 2007; Bernardi, 2007; Tegtbur, 2003). However, Pierce, 2007 and Braith, 2007 presented a big loss of follow up (> or equal 20%). There was no bliding, even for statistic analysis in all papers included. The other included studies presented nor serious risk of bias (Haykowsky, 2009, Kobashigawa, 1999, Monk-Hansen, 2013, Rustad, 2014, Nytroen, 2012, Hermann, 2011).
i. Indirectness can be related to differences in intervention, in outcomes or population. Even considering differences in training modalities, (both aerobic and combined training included), training intensities (HIIT and moderate intensity) and some differences in regards the cardiopulmonary exercise testing methodology, the authors believe there is no reason to rate down for indirectness. This because none of those criteria were able to affect the overall comparison for VO2 peak.
j. Considering not dicotomic outcomes, all data from imprecision were based on confidence intervals and sample size. Even considering the variations on confidence intervals for some studies, the authors did not consider it important for the main result.
k. Indirectness can be related to differences in intervention. Among the 4 included studies, 2 applied aerobic intervention (Nitroen 2012, Pascoalino 2015), while 2 applied combined intervention, resistance and aerobic exercises (Haykowsky, 2009 and Kobashigawa, 1999), however, since it is already included as subgroup analysis the authors decided not rate down for variation, but considering the differences in aerobic interventions (HIIT and moderate continuous training, Nitroen, 2012 and Pascoalino, 2015, respectively), the authors rate down for indirectness.
l. Considering not dicotomic outcomes, all data from imprecision were based on confidence intervals. Excepting Nitroen, 2012, all studies included included negative values in their confidence interval.
m. There was 1 study included with high risk of bias due to unclear randomization and allocation concealed procedure further 30% of loss to follow-up (Pierce, 2007). Also, Wu, 2008 and Pascoalino,2014 scored as serious risk of bias also due to unclear randomization and allocation concealed procedure. No blinding were considered for data analysis in any study. The authors judged Kobashigawa, 1999, Haykowsky, 2009, Monk-Hansen, 2013, Nytroen, 2012 and Rustad, 2014 as not serious risk of bias.
n. All data from imprecision were based on confidence interval and sample size. Considering 4 of the included studies crossed the neutral line and 3 did not crossed it, the authors decided to classify imprecision as serious.
o. Although points estimates did not vary widely across studies and has showed overlap, the authors rated down for insonsistency due to high heterogeneity.
p. All data from imprecision were based on confidence interval and sample size. The main concern for this outcome is the general low sample size. Also, although Wu, 2008 indicated no problems regarding confidence interval, Kobashigawa 1999 presented results in both directions of the forest plot.
q. Considering only two studies were included for this outcome and the poin estimates vary widely across them, the authors rated down for inconsistency.
r. All studies presented positive and negative values in theis minimum and maximun confidence interval.

Supplementary Figure S3: Summary of Findings Tables with quality of evidence for HIIT compared to moderate continous training.

Author(s):
Question: What is the certainty of the evidence for the comparison in between HIIT and MCT-AE after Heart Transplantation?
Setting: Exercise Training in Heart Transplantation
Bibliography: Turri-Silva et al, 2020

Certainty assessment							№ of patients		Effect		Certainty	Importance
№ of studies	Study design	Risk of bias	Inconsistency	Indirectness	Imprecision	Other considerations	HIIT	MCT-AE	Relative (95% CI)	Absolute (95% CI)		
**VO2 peak**												
2	randomised trials	not serious	not serious	serious [a]	not serious	none	53	57	-	MD **1.96 higher** (0.99 higher to 2.93 higher)	⨁⨁⨁◯ MODERATE	

CI: Confidence interval; **MD:** Mean difference

Explanations

a. There are some differences in regards the training prescription. On Nytroen 2019 the HIIT intervention took longer than Dall 2014. Also, HIIT was not the only kind of exercise on Nytroen 2019, since the authors also included resistance training sections during some months. So, the evidence is not only direct provenient from HIIT protocols.
